# Immunoinformatics analysis of *Brucella melitensis* to approach a suitable vaccine against brucellosis

**DOI:** 10.1186/s43141-023-00614-6

**Published:** 2023-11-29

**Authors:** Pejman Hashemzadeh, Saba Asgari nezhad, Hossein Khoshkhabar

**Affiliations:** 1 Department of Medical Biotechnology, School of Medicine, Lorestan University of Medical Sciences, Khorramabad, Lorestan Iran; 2 Department of Immunology, School of Medicine, Lorestan University of Medical Sciences, Khorramabad, Lorestan Iran

**Keywords:** Multi-epitope vaccine, Brucellosis, Immunoinformatics, Epitope prediction

## Abstract

**Background:**

Brucellosis caused by *B. melitensis* is one of the most important common diseases between humans and livestock. Currently, live attenuated vaccines are used for this disease, which causes many problems, and unfortunately, there is no effective vaccine for human brucellosis. The aim of our research was to design a recombinant vaccine containing potential immunogenic epitopes against *B. melitensis*.

**Methods:**

In this study, using immunoinformatics approaches, 3 antigens Omp31, Omp25, and Omp28 were identified and the amino acid sequence of the selected antigens was determined in NCBI. Signal peptides were predicted by SignaIP-5.0 server. To predict B-cell epitopes from ABCpred and Bcepred servers, to predict MHC-I epitopes from RANKPEP and SYFPEITHI servers, to predict MHC-II epitopes from RANKPEP and MHCPred servers, and to predict CTL epitopes were used from the CTLPred server. Potentially immunogenic final epitopes were joined by flexible linkers. Finally, allergenicity (AllerTOP 2.0 server), antigenicity (Vaxijen server), physicochemical properties (ProtParam server), solubility (Protein-sol server), secondary (PSIPRED and GRO4 servers) and tertiary structure (I-TASSER server), refinement (GalaxyWEB server), validation (ProSA-web, Molprobity, and ERRAT servers), and optimization of the codon sequence (JCat server) of the structure of the multi-epitope vaccine were analyzed.

**Results:**

The analysis of immunoinformatics tools showed that the designed vaccine has high quality, acceptable physicochemical properties, and can induce humoral and cellular immune responses against *B. melitensis* bacteria. In addition, the high expression level of recombinant antigens in the *E. coli* host was observed through in silico simulation.

**Conclusion:**

According to the results in silico, the designed vaccine can be a suitable candidate to fight brucellosis and in vitro and in vivo studies are needed to evaluate the research of this study.

## Background

*Brucella* are facultative intracellular and gram-negative coccobacillus bacteria, which causes brucellosis in a variety of animal species and humans. *Brucella melitensis*, *Brucella abortus*, and *Brucella suis* are the maximum malignant species in order of pathogenicity [1].

Brucellosis is a global zoonotic disease which endemic in plenty countries, including the Latin America, Africa, central Asia, and Middle East [2]. This disease not only causes problems for humans, but also causes great damage to livestock and agriculture. Features of the disease with chronic infections with symptoms are such as arthritis, undulating osteomyelitis, and fever in humans, as well as with reduced fertility and abortion in animals [3–5].

Therefore, it is very important to strengthen the diagnosis and treatment of this disease [6, 7]. Accordingly, a novel vaccine aimed at stimulating the immune system’s response has a high potential for success. But unfortunately, an effective and no side effects vaccine for human brucellosis is not available yet [8, 9]. All available animal vaccines are based on live weakened strains of *Brucella* (Rev1, S19, and RB51), which can be potentially effective but are infectious to humans and cause abortion in pregnant animals [10, 11].

Due to the limitations and disadvantages of live, attenuated vaccines, a new treatment method is needed. Peptide vaccines based on multiple epitopes have advantages compared to live attenuated vaccines, because the production of these novel vaccines is relatively fast and cheap, and they are also safe for the host [12, 13]. Research has shown that multi-epitope vaccines containing immune cell epitopes are able to stimulate protective immunity against different diseases [14–16]. A useful peptide-based vaccine should include T-cell epitopes and B-cell epitopes to stimulate numerous humoral and cellular immunity system response [17–19].

The clue to important brucellosis diagnosis and treatment is to find efficient antigen. Hence, the development of efficient antigens is crucial for improving performance the immune system response of brucellosis multi-epitope vaccines and the sensitivity and specificity of diagnosis and treatment methods [20, 21].

Nevertheless, the recognition of antigenic epitopes as novel vaccine candidates for designing recombinant vaccines is a costly and time-consuming method. Recently, due to the advances in structure and immunoinformatics vaccinology, the prediction of epitopes for T-lymphocytes and B-lymphocytes helps researchers to identify specific peptides [22, 23]. In this work, using previous studies and immunoinformatics tools, three antigens for the new recombinant vaccine against brucellosis including 31 kDa outer membrane protein (Omp31), outer membrane protein 25 (Omp25), and outer membrane protein 28 (Omp28) were selected and evaluated. Antigens in the outer membranes of bacteria (OMPs) play an essential role in stimulating the immune system [24]. The characteristics of selected antigens are as follows:

Omp31 is a play an essential role in conferring defense against *Brucella* and main membrane protein of *Brucella*. This antigen is a significant virulence factor of *Brucella* and is of high potential as an antigenic protein for vaccination [25].

Studies have shown that Omp25 protein is highly conserved in different species of *Brucella*. Omp25 is highly immunogenic and leads to an inflammatory response in brucellosis by activating MAPK signaling pathway [26].

Researchers use suitable adjuvants to induce effective immunity in the vaccine. Adjuvants can increase the immunogenicity of antigens and create effective and long-lasting immune responses [27, 28]. Therefore, Omp28 protein was used. Omp28 is considered as an important outer membrane protein of *Brucella* and it has also been reported that Omp28 peptide with CpG oligonucleotide as an adjuvant can induce an immune response, indicating that this protein stimulates the immune system [29].

Immunoinformatics or computational immunology is the interface between computer science and experimental immunology, which represents the use of computational methods and resources to understand immunological information [30]. Therefore, to confirm the availability of the recombinant structure, the physicochemical properties, allergenicity, antigenicity, solubility, secondary, and tertiary structures of the vaccine were analyzed with different immunoinformatics servers. The results showed that the designed recombinant construct can be used to fight *B. melitensis*.

## Methods

**Fig. 1:**
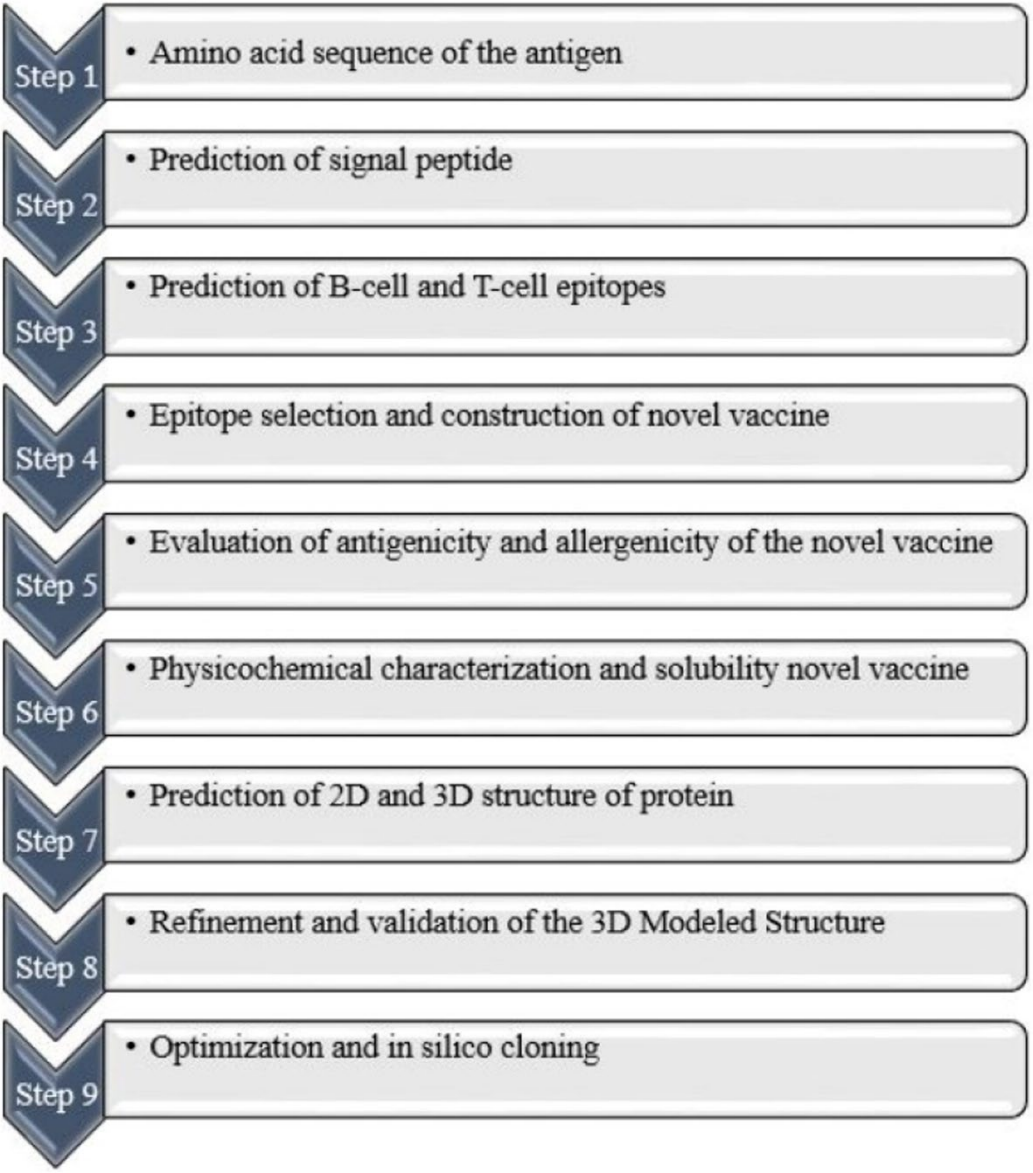
Schematic representation of multi-epitope vaccine design against *B. melitensis*

Summarized steps of the designing multi-epitope vaccine are presented as flowchart shown in Fig. 1.

### Sequence analysis

The complete amino acid sequences of Omp31 (accession ACS50328.1), Omp25 (accession AEF59022), and Omp28 (accession AEF59021) were retrieved from the National Centre for Biotechnology Information (NCBI) at www.ncbi.nlm.nih.gov in FASTA format [31].

### Prediction of signal peptide

The SignalP-5.0 server (https://services.healthtech.dtu.dk/service.php?SignalP-5.0) provides a resource for predicting signal peptide cleavage sites in proteins. This server uses convolutional and recurrent (LSTM) neural networks to identify sequence motifs of different lengths [32].

### Prediction of B-cell epitopes

The B-cell epitopes of Omp31, Omp25, and Omp28 antigens were predicted using ABCpred (http://crdd.osdd.net/raghava/abcpred/) and Bcepred (http://crdd.osdd.net/raghava/bcepred/) servers [33, 34]. Prediction of B-cell epitopes in ABCpred server using artificial neural network technique [35]. Bcepred server to predict B-cell epitopes using physico-chemical properties. The results of Bcepred server are presented in graphic and tabular format. This server predicts epitopes with 58.7% accuracy and 2.38 threshold [36].

### Prediction of MHC-I epitopes

The MHC-I epitopes of Omp31, Omp25, and Omp28 antigens were predicted by RANKPEP (http://imed.med.ucm.es/Tools/rankpep.html) and SYFPEITHI (http://syfpeithi.de/) server [37]. The RANKPEP servers rank all possible epitopes from an input antigen using position-specific scoring matrices (PSSM). MHC-I-restricted epitopes are predicted at a threshold of 2–3% of high-scoring peptides [38]. SYFPEITHI server uses binding status scoring qualitative prediction methods for T-cell epitope prediction [39].

### Prediction of MHC-II epitopes

The MHC-II epitopes of Omp31, Omp25, and Omp28 antigens were predicted by MHCPred V.2.0. (http://www.ddg-pharmfac.net/mhcpred/MHCPred/) and RANKPEP (http://imed.med.ucm.es/Tools/rankpep.html) servers. The RANKPEP server ranks all possible epitopes from an input antigen using position-specific scoring matrices (PSSM). MHC-II-restricted epitopes are predicted at a threshold of 4–6% of high-scoring peptides [38]. MHCPred uses an additive method to predict MHC-II epitopes as well as transporter-associated processing (TAP). The order of epitopes is arranged based on IC50 values. If the IC50 value is higher than 5000, the peptide does not bind to MHC molecules. Therefore, epitopes with lower IC50 values are selected [40].

### Prediction of CTL epitopes

The CTL (cytotoxic T lymphocyte) epitopes of Omp31, Omp25, and Omp28 antigens was predicted using CTLPred (http://crdd.osdd.net/raghava/ctlpred/applying) server. This server predicts CTL epitopes based on artificial neural network (SVM) and support vector machine (ANN). CTLpred server accuracy is 75.8% [41].

### Epitope selection and construction of novel vaccine

The results were compared and regions with the highest score and with the maximum overlap that induce the cellular and humoral immune responses were identified and finally the selected epitopes were joined by linkers.

#### Linkers

The final selected epitopes need a certain degree of flexibility; in this research, two flexible linkers (GSGSGS and GGGGS) were used to connect the epitopes.

### Evaluation of antigenicity and allergenicity of the novel vaccine

Recombinant vaccine was experiment for antigenicity using VaxiJen v2.0 server (http://www.ddg-pharmfac.net/vaxijen/VaxiJen/VaxiJen.html) with a threshold value ≥ 0.4. This server’s approach for antigen prediction is based on auto cross covariance (ACC) transformation of protein sequences into uniform vectors with amino acid features [42]. Allertop v2.0 server (https://www.ddg-pharmfac.net/AllerTOP/) with threshold value of − 0.4 was used to prevent allergic reactions to recombinant vaccine epitopes. The approach of this server is based on converting ACC of protein sequence into uniform vectors of equal length [43].

### Physicochemical characterization and solubility novel vaccine

The ProtParam server) https://web.expasy.org/protparam/ (generates physico-chemical parameters for an amino acid sequence. The computed parameters include the grand average of hydropathicity (GRAVY), instability index, aliphatic index, extinction coefficient, atomic composition, estimated half-life, amino acid composition, theoretical pI, and molecular weight [44].

The Protein-sol server (https://protein-sol.manchester.ac.uk/) was used to analyze the solubility of the recombinant antigen. This server is a web based suite of theoretical calculations and predictive algorithms for understanding protein solubility and stability [45].

### Protein secondary structure prediction

PSIPRED web server (http://bioinf.cs.ucl.ac.uk/psipred/) was used to predict the second structure. This server uses a very stringent cross-validation method to evaluate the performance of the method. The Q3 score averages 81.6% [46].

GOR IV server (https://npsa.lyon.inserm.fr/cgi-bin/npsa_automat.pl?page=/NPSA/npsa_gor4.html) was used to predict secondary structure novel multi-epitopes construct [47].

### Protein tertiary structure prediction

I-TASSER server (https://zhanggroup.org/I-TASSER/) was used to predict the 3D structure and function of recombinant antigen. This server uses a hierarchical method for prediction. The evaluation parameter of this server to choose the best model is based on C-score. C-score is a confidence score for estimating the quality of the predicted 3D structures; the score range of this server is (− 5, 2), where a C-score with a higher score indicates a higher quality model [48, 49].

### Refinement of the 3D modeled structure

GalaxyRefine server (http://galaxy.seoklab.org/cgi-bin/submit.cgi) is used to refine the best 3D model of I-TASSER server. This server provides more accurate structures for determining the experimental structure, functional study, and molecular design [50].

### Validation of the 3D modeled structure

Three-dimensional refined recombinant vaccine structures were validated by ProSAweb, Molprobity, and ERRAT servers. The ProSAweb server at https://prosa.services.came.sbg.ac.at/prosa.php calculates the overall quality score by checking it out the atomic peculiarities of the model. The ProSA-web *z*-score is depicted on a plan, which contains the *z*-score of the experimental structures entrusted in PDB. This server only needs Cα atoms to evaluate low-resolution structures [51]. Molprobity server (http://molprobity.biochem.duke.edu) was used for the Ramachandran plot [52]. Calculate phi/psi angles (ϕ, ψ) for any antigenic protein residue, and the residuals include favored regions, allowed regions, and outlier regions [53]. Also, the ERRAT tool (https://saves.mbi.ucla.edu/) was used. This tool is used to verifying protein structures determined by crystallography [54].

### Optimization and in silico cloning

The amino acid sequence of the recombinant vaccine was converted to DNA sequence (Reverse Translate) using SMS (Sequence Manipulation Suite) server (https://www.bioinformatics.org/sms2/) [55]. The JCAT server (http://www.jcat.de/) was used to optimize and increase the expression (avoiding cleavage sites for restriction enzymes, predicting GC content, and codon adaption index score (CAI) of the recombinant vaccine protein in the *E. coli* K12 system [56]. In the end, to clone the recombinant vaccine sequence in the pET-26b vector, HindIII and BamHI restriction sites were added for the N and C terminal of the sequence, respectively.

## Results

### Sequence analysis

In the present study, Omp31, Omp25, and Omp28 proteins with 240, 213, and 250 amino acids, respectively, were selected as immunogenic proteins for recombinant vaccine design. The sequences of these proteins were obtained from the NCBI database.

### Prediction of signal peptide

**Fig. 2:**
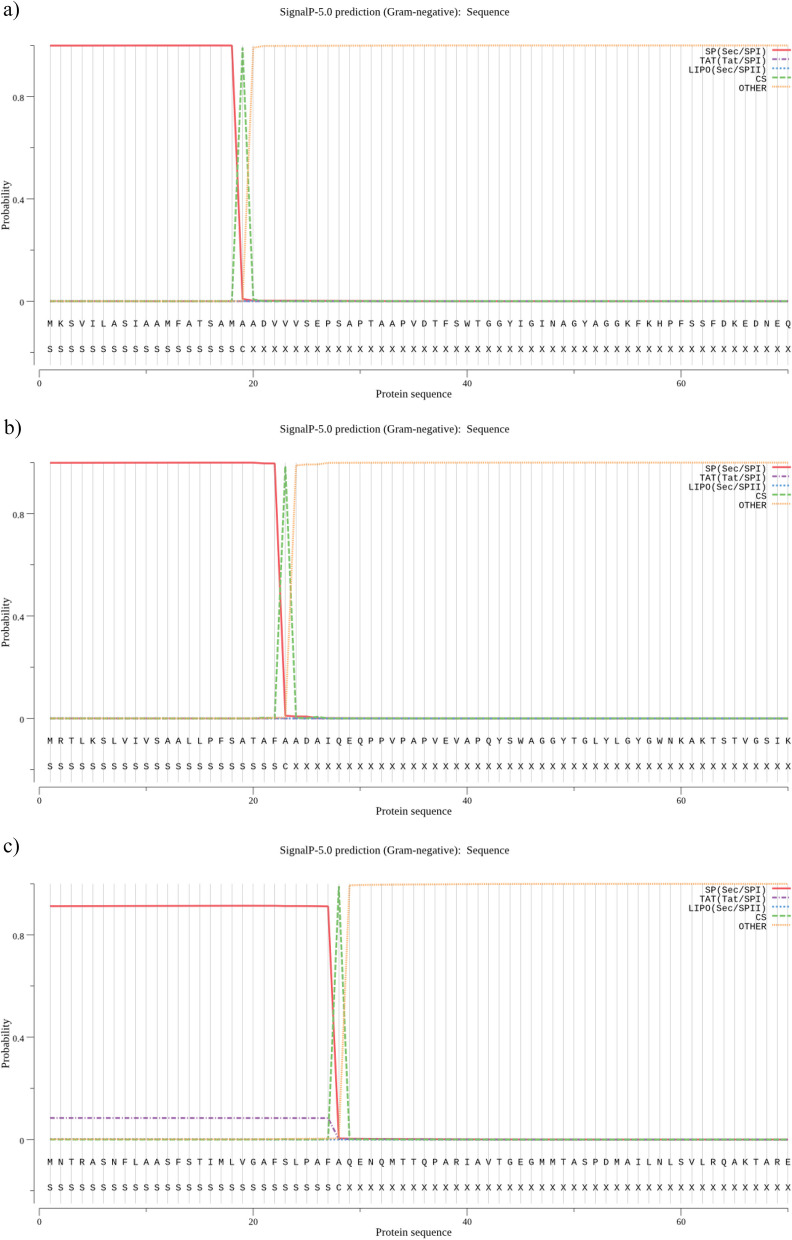
By SignalP-5.0 server, the peptide signal of selected antigens was checked. SP (Sec/SPI), type of signal peptide predicted; CS, the cleavage sits; Other, the probability that the sequence does not have any kind of signal peptide. The signal peptide sequence of Omp31 (**a**), Omp25 (**b**), and Omp28 (**c**) was MKSVILASIAAMFATSAMA, MRTLKSLVIVSAALLPFSATAFA, and MNTRASNFLAASFSTIMLVGAFSLPAFA, respectively

SignalP-5.0 server was used to predict the signal peptides of Omp31, Omp25, and Omp28 proteins. The signal peptide sequence of Omp31, Omp25, and Omp28 was MKSVILASIAAMFATSAMA, MRTLKSLVIVSAALLPFSATAFA, and MNTRASNFLAASFSTIMLVGAFSLPAFA, respectively (Fig. 2). The signal peptide sequences were removed from the epitope prediction of the 3 selected proteins.

### Prediction of B-cell epitopes

**Fig. 3:**
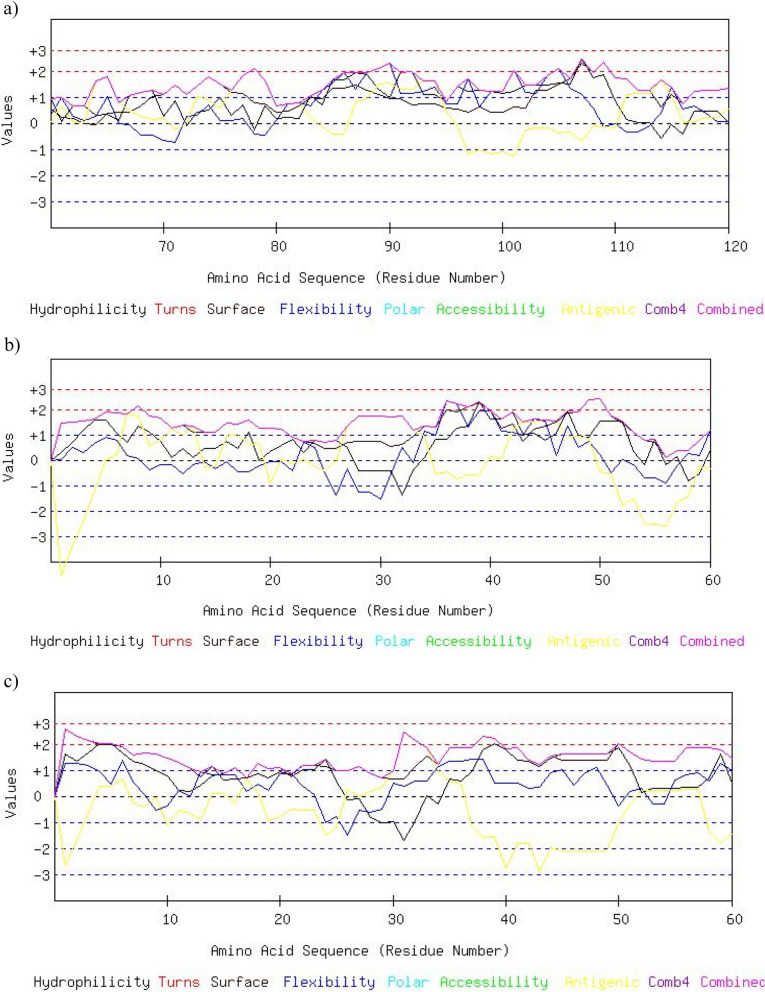
B-cell epitopes of Omp31 (**a**), Omp25 (**b**), and Omp28 (**c**) antigens were predicted using Bcepred server. The results of this server are shown as graphical result based on features such as hydrophilicity, flexibility, antigenic propensity, and exposed surface

Three selected antigens were predicted B-cell epitopes using Bcepred server. The amino acid sequence was analyzed based on properties such as hydrophilicity, flexibility, antigenic propensity, and exposed surface of this server. The results of this server are shown as graphical result and overlap display in Fig. 3.

To further ensure the obtained results, ABCpred server was used to predict B-cell epitopes. The results of this server are shown in Table 1.

**Table 1:** Results of prediction of B-cell epitopes using ABCpred server are shown

Antigen	Sequence	Start position	Score
Omp31	YIGINAGYAGGKFKHP	25	0.93
	LHTWSDKTKAGWTLGA	155	0.90
	GWTLGAGAEYAINNNW	165	0.89
	TGSISAGASGLEGKAE	93	0.88
	VVSEPSAPTAAPVDTF	4	0.86
Omp25	QDQIVYGVEGDAGYSW	63	0.94
	GWTAGAGLEAKLTDNI	135	0.93
	TGLYLGYGWNKAKTST	27	0.91
	AGIAGSQIKLNNGLDD	113	0.91
	RVEYRYTQYGNKNYDL	153	0.89
Omp28	MPMPIARGQFRTMLAA	181	0.89
	QPIYVYPDDKNNLKEP	76	0.88
	GEGMMTASPDMAILNL	16	0.88
	LGVNQGGDLNLVNDNP	122	0.88
	PARIAVTGEGMMTASP	9	0.87

### Prediction of MHC-I epitopes

RANKPEP and SYFPEITHI servers were used to predict MHC-I epitopes of antigens. The results of SYFPEITHI and RANKPEP servers for selecting the best epitopes of Omp31, Omp25, and Omp28 antigens are shown in Table 2.

**Table 2:** The prediction results of MHC-I epitopes using RANKPEP and SYFPEITHI servers are shown

Server	Antigen	Sequence	Start position	Score
RANKPEP	Omp31	YAINNNWTL	174	20.94
		AINNNWTLK	175	17.59
		WQLDNGVVL	73	16.82
		YIGINAGYA	25	11.45
	Omp25	YGNKNYDLA	161	21.78
		FAGWNFQQD	56	19.94
		GYGWNKAKT	32	8.52
		DLNPVMPYL	103	5.34
	Omp28	MAILNLSVL	26	20.64
		TGGINIQPI	70	17.99
		AAPDNSVPI	196	13.39
		AAGENSYNV	205	12.14
SYFPEITHI	Omp31	YAINNNWTL	174	27
		WQLDNGVVL	73	25
		LGKRNLVDV	191	21
		ESKVNFHTV	205	21
	Omp25	FAGWNFQQD	56	18
		AGGYTGLYL	23	17
		VMPYLTAGI	107	16
		TAGIAGSQI	112	16
	Omp28	AAPDNSVPI	196	30
		MAILNLSVL	26	28
		AMTANNEAM	43	25
		TGGINIQPI	70	25

### Prediction of MHC-II epitopes

MHCPred V.2.0. and RANKPEP servers were used to predict MHC-II epitopes of antigens. The results of MHCPred V.2.0. and RANKPEP servers for selecting the best epitopes of Omp31, Omp25, and Omp28 antigens are shown in Table 3.

**Table 3:** The prediction results of MHC-II epitopes using RANKPEP and MHCPred servers are shown

RANKPEP	Antigen	Sequence	Start position	Score
	Omp31	VEWFGTVRA	111	10.84
		KVEWFGTVR	110	7.57
		VTAGGFVGG	58	6.12
		GGFVGGVQA	61	3.29
	Omp25	GSLRARVGY	94	8.16
		QDFRVGIGY	180	7.45
		RARVGYDLN	97	3.96
		VMPYLTAGI	107	2.76
	Omp28	RGQFRTMLA	187	20.51
		VRELANVGK	106	15.91
		VVEISELSR	170	14.60
		ISELSRPPM	173	8.42
MHCPred	Antigen	Sequence	Start position	Predicted -logIC50 (M)
	Omp31	YNWQLDNGV	71	7.537
		FDKEDNEQV	44	7.531
		VDNSFLESK	199	7.52
		YLYTDLGKR	186	7.5
	Omp25	KLDTQDFRV	176	7.42
		YDLNPVMPY	102	7.41
		DLNPVMPYL	103	7.35
		YSWAGGYTG	20	7.25
	Omp28	GENSYNVSV	207	7.4
		GMMTASPDM	18	7.3
		KNNLKEPTI	85	7.2
		NLVNDNPSA	131	7

### Prediction of CTL epitopes

The CTLPred server was used to predict the potential CTL epitopes. The results of this server for Omp31, Omp25, and Omp28 antigens are shown in Table 4.

**Table 4:** Results of prediction of CTL epitopes using CTLPred server are shown

Antigen	Sequence	Start position	Score
Omp31	AGAEYAINNN	170	1.000
	EPSAPTAAP	7	0.990
	YTATERLMV	123	0.990
Omp25	APVEVAPQY	12	0.990
	PQYSWAGGY	18	0.990
	GWTAGAGLE	135	0.990
Omp28	DNPSAVINE	135	1.000
	RKRAVANAI	145	1.000
	DMAILNLSV	25	0.990

### Epitope selection and construction of novel vaccine

**Fig. 4:**
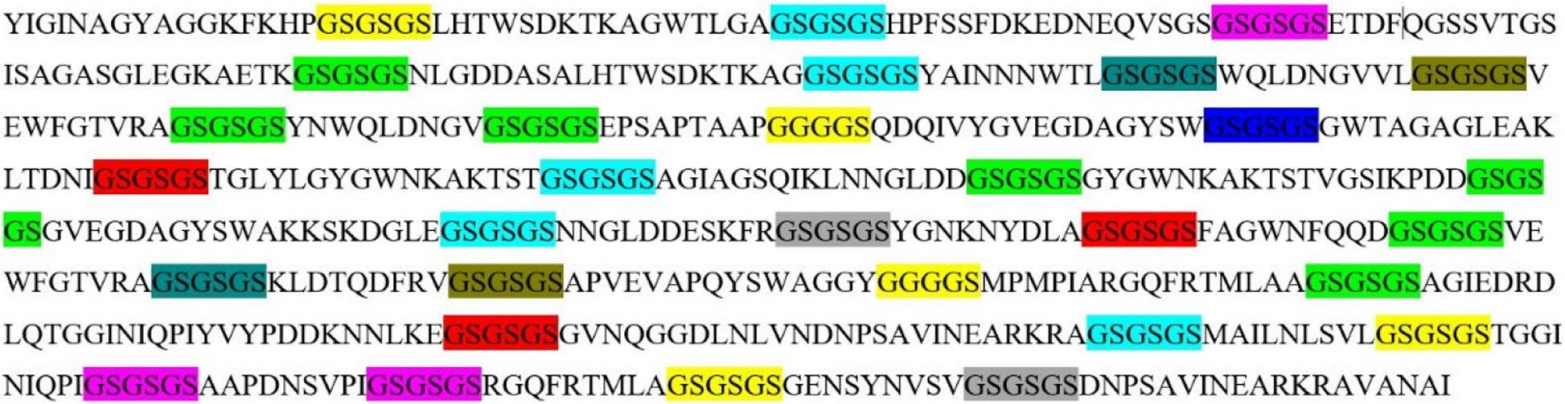
A representation of the amino acid sequence of the recombinant vaccine

Based on B-cell, MHC-I, MHC-II, and CTL epitopes, which have high overlap and good score, thirty-one epitopes of Omp31, Omp25, and Omp28 antigens were selected as the final regions. The epitopes selected from each antigen were fused by GSGSGS and GGGGS linkers. Finally, the recombinant sequence including epitopes and linkers is shown in Fig. 4.

### Evaluation of antigenicity and allergenicity of the novel vaccine

The results obtained by the AllerTOP 2.0 server showed that the designed vaccine is non-allergenic. The probability of antigenicity of the vaccine was predicted by the Vaxijen server to be 1.4616% at a threshold of 0.4%, which means that our vaccine can stimulate the immune system.

### Physicochemical characterization and solubility novel vaccine

**Fig. 5:**
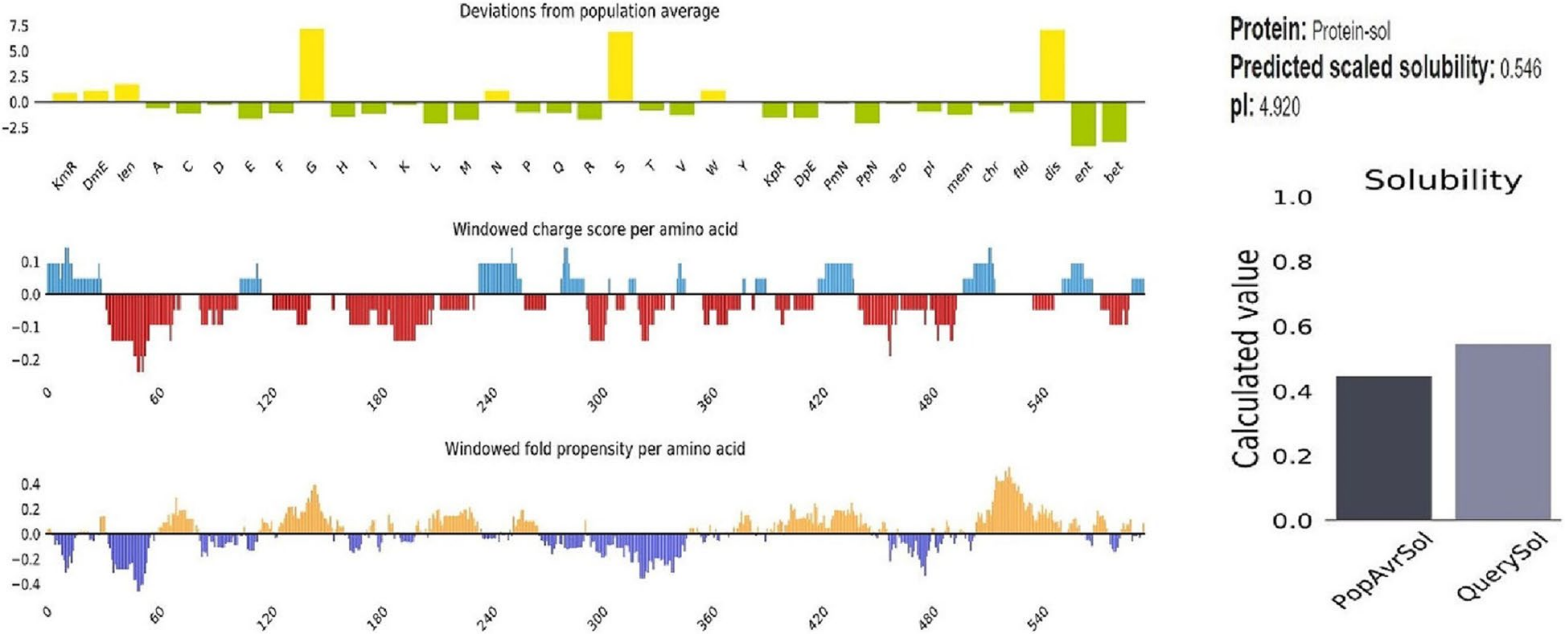
The analysis of the Protein-sol server showed that the target recombinant vaccine is soluble. The solubility of the vaccine construct was shown to be 0.546 compared to 0.45 of the population average solubility of *E. coli*

The results of ProtParam and protein-sol servers are shown in Table 5. Analysis of the ProtParam server showed that the recombinant vaccine could be classified as a stable protein. The negative value of the GRAVY (grand average of hydropathy) indicates the non-polarity of this recombinant protein. The high value of the aliphatic index of the recombinant protein showed that protein is thermo-stable over a wide temperature range. The analysis of the protein-sol server showed that the target protein is soluble (Fig. 5).

**Table 5:** Analysis of physicochemical properties of recombinant vaccine

Physicochemical properties	Result
Number of amino acids	618
Molecular weight	60079.77
Theoretical Pi	4.920
Total number of negatively charged residues (Asp + Glu)	51
Total number of positively charged residues (Arg + Lys)	39
Total number of atoms	8136
instability index (II)	18.75
Aliphatic index	51.36
Grand average of hydropathicity (GRAVY)	− 0.517
Solubility	0.546

### Protein secondary structure prediction

**Fig. 6:**
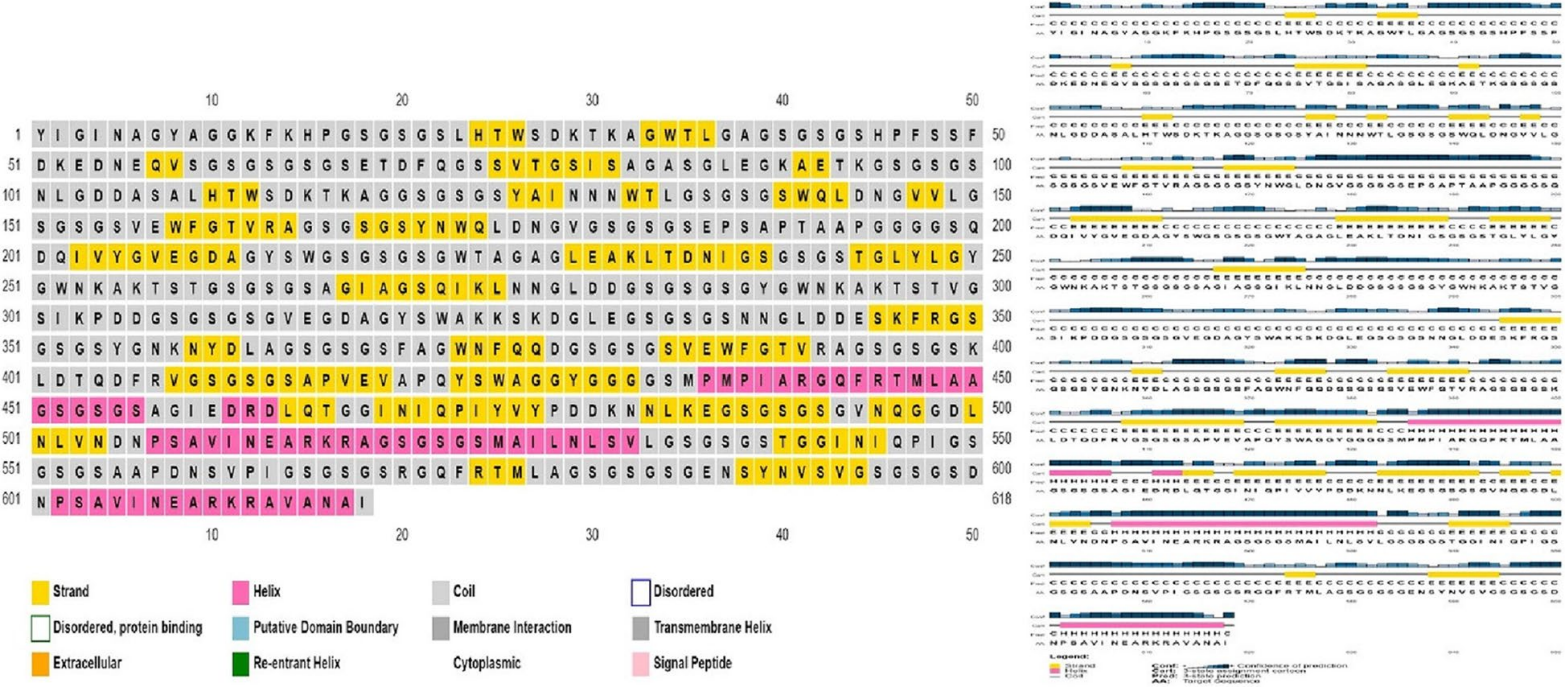
Graphic representation of the characteristics of the second structure of the recombinant vaccine. Alpha-helix residues, coil residues, and beta-strand residues are shown in pink, gray, and yellow, respectively

**Fig. 7:**
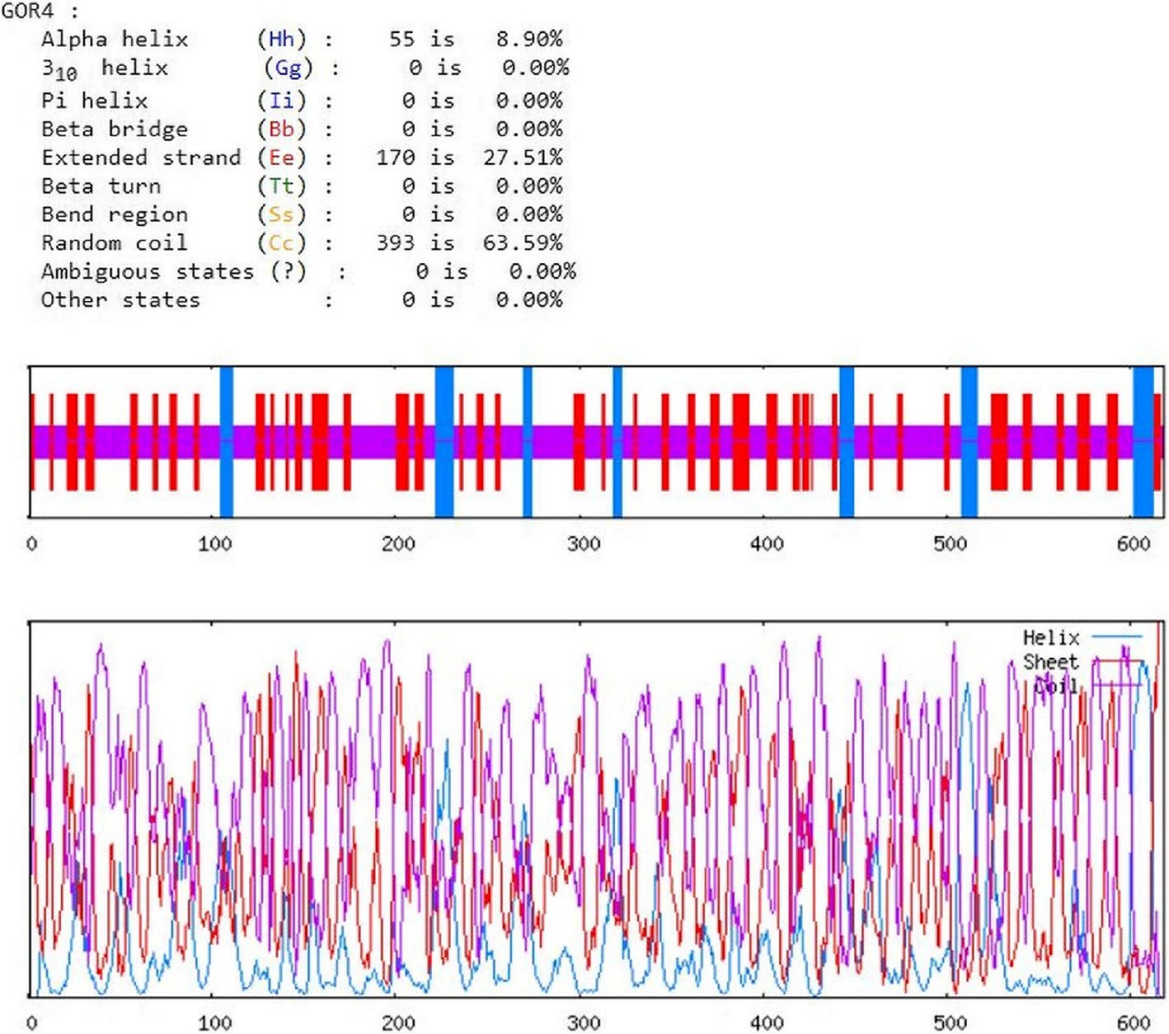
Secondary structure specifications are shown by the GRO4 server. Alpha helixs, extended strands, and beta turns are shown in blue, red, and green, respectively

The graphical representation of secondary structure specification by PSIPRED server is shown in Fig. 6. Secondary structure specifications result by GRO4 server included 27.51% extended strand, 8.90% alpha helix, and 63.59% random coil (Fig. 7).

### Protein tertiary structure prediction

**Fig. 8:**
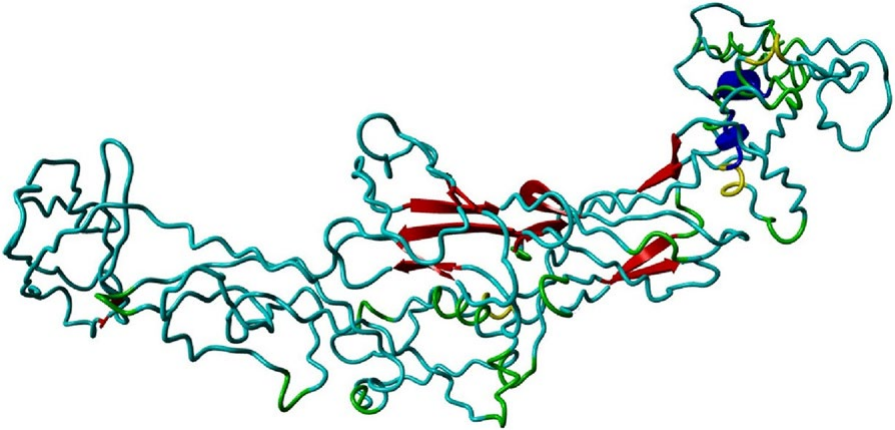
The 3D model of the vaccine construct obtained after homology modeling on I-TASSER

Five models were predicted by I-TASSER server for tertiary structure. The predicted models had C-score values in the range of − 1.43 to − 3.59. Since the C-score with a higher score indicates a higher quality model, then model one with a C-score of − 1.43 was selected (Fig. 8).

### Refinement and validation of the 3D modeled structure

**Fig. 9:**
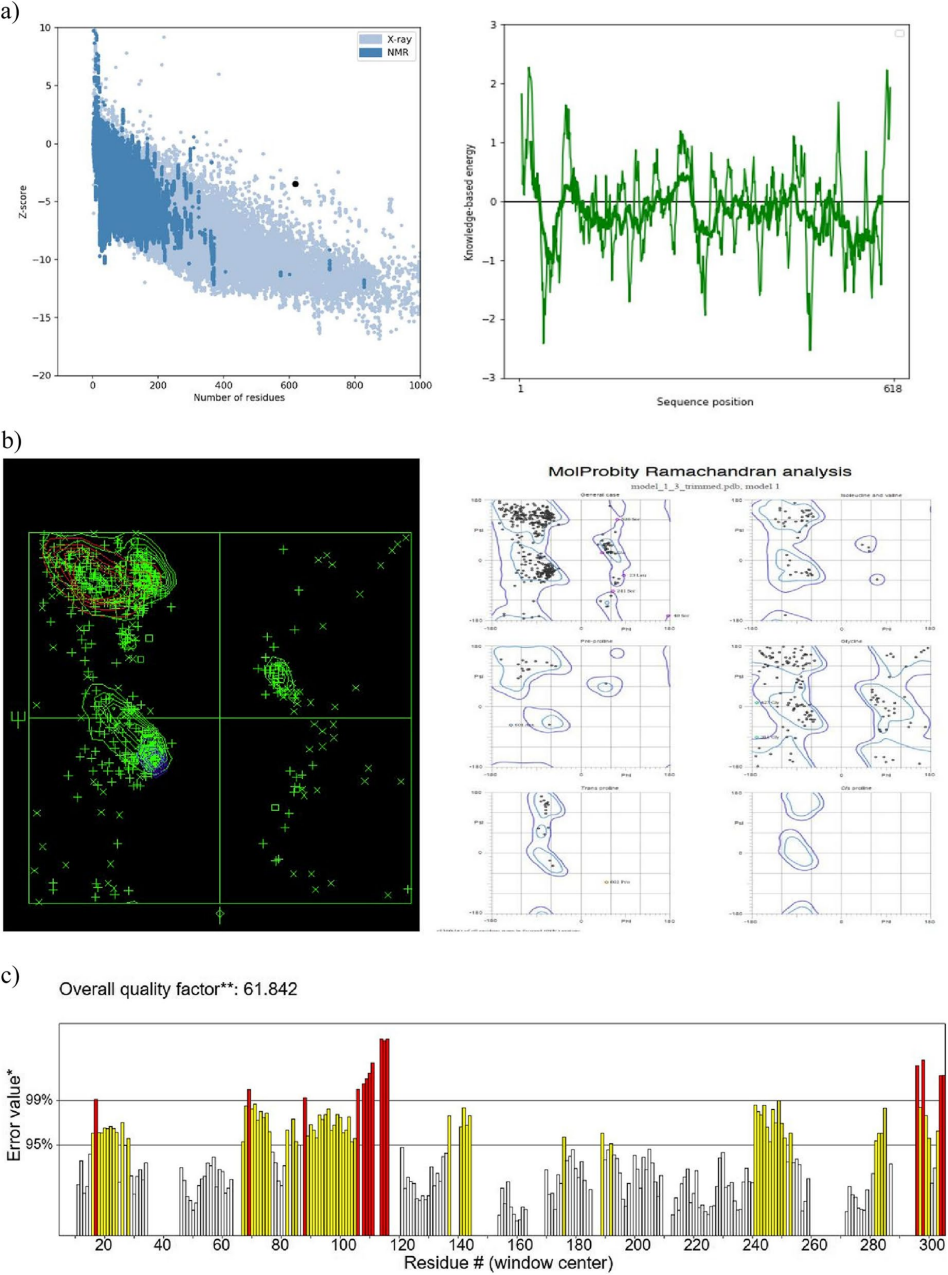
**a** The *z*-score value of the recombinant vaccine is − 3.47, which was obtained by the ProSA-web server. **b** The 3D structure of the purified recombinant vaccine was evaluated by Molprobity server: 79.3% residues in most favored regions, 17.0% residues in additional allowed regions, 1.3% residues in generously allowed regions, and 2.4% residues in disallowed regions. **c** The quality score value of the recombinant vaccine is 61.842%, which was obtained by the ERRAT server

The predicted 3D structure is refined by the GalaxyWEB server. The refined model was validated by ProSA-web, Molprobity, and ERRAT servers. The ProSA-web server was checked for the recombinant vaccine and the *z*-score value of − 3.47 was provided (Fig. 9a). The Ramachandran plot for the recombinant vaccine was examined and showed that 79.3% residues in most favored regions, 17.0% residues in additional allowed regions, 1.3% residues in generously allowed regions, and 2.4% residues in disallowed regions (Fig. 9b). The ERRAT tool showed a quality score of 61.842% (Fig. 9c).

### Optimization and in silico cloning

**Fig. 10:**
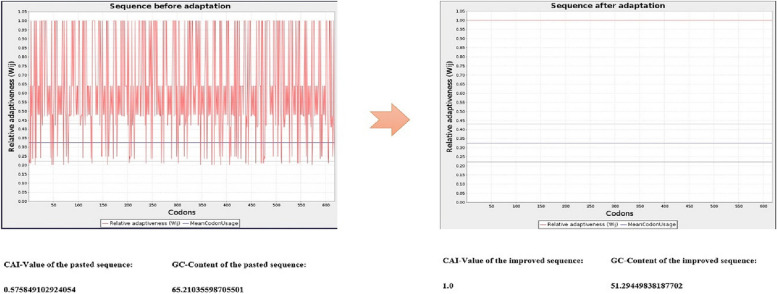
The results of JCat server improved the nucleotide sequence of the recombinant vaccine and changed the GC-content to about 51.29% and CAI value of 1.0

The JCat server improved the nucleotide sequence of the recombinant vaccine and changed the CAI-value of the pasted sequence from 0.5 to 1.0 and the GC-content of the pasted sequence from 65.21 to 51.29%. These results indicate the excellent potential of high expression of the recombinant vaccine in the *E. coli* host (Fig. 10).

## Discussion

Brucellosis is one of the common diseases between humans and animals, which is associated with fever in humans and abortion in animals [57]. Approximately 500,000 cases of brucellosis are reported annually worldwide. The spread of this disease is increasing due to tourist trips, trade, and immigration [58]. Lack of effective human vaccine, long treatment period, relatively low efficiency of animal vaccines, and the process of prevention and treatment of this disease have faced serious problems. Accordingly, there is an urgent need for new approaches or effective recombinant vaccines to fight against *Brucella* [59, 60].

Currently, human vaccine against this disease is not available and the vaccines that are used are live attenuated vaccines to immunize animals, but these vaccines have problems such as abortion in livestock, disease in humans, and production of antibodies that make it difficult to diagnose infected animals from vaccinated animals [61].

Therefore, trying to produce an effective vaccine to prevent this infection is inevitable. Studies show that in vaccine design, the use of several different antigens in the vaccine composition can be more effective in its performance [62, 63]. In this research, we used different immunoinformatics tools to design a multi-epitope recombinant construct. According to previous research, Omp31, Omp25, and Omp28 antigens were selected for use in this study, because they are highly conserved and effective in creating an immune response [64, 65].

In the first step of this study, using in silico tools, we identified the immunogenic epitopes of 3 antigens and removed the signal peptide sequences from the final antigen sequences.

Previous studies show that in order to increase the effectiveness of *Brucella* vaccines, they should stimulate humoral and cellular immune responses [66]. Therefore, the second step in this study is to predict the potential epitopes of B cells, MHC-I, MHC-II, and CTL, which were predicted by online servers (ABCpred, Bcepred, RANKPEP, SYFPEITHI, MHCPred, and CTLPred). Then, the epitopes with the highest score and the most overlap (thirty-one epitopes) were selected and fused together by flexible linkers (GSGSGS and GGGGS). Linkers are an important component of multi-epitope fusion antigens, increase stability in the structure of recombinant antigens, and improve biological activity and increase expression efficiency [67].

The third step of this study was the examination of the immunological characteristics and the physical and chemical characteristics of the recombinant construct, which was analyzed based on immunoinformatics methods. The obtained results showed that the recombinant construct is a strong antigen and non-allergenic and had an acceptable solubility percentage. The results of physicochemical properties by ProtParam tool showed that this multi-epitope protein is classified as a stable protein.

The fourth step of this study was to investigate the secondary and tertiary structure of the recombinant vaccine. Studies on secondary structures showed 27.51% extended strand, 8.90% alpha helix, and 63.59% random coil. The results of the three-dimensional structure of the recombinant vaccine by the I-TASSER server showed that model 1 is the best model to choose among the 5 models, because model 1 has an estimated TM score of 0.54 ± 0.15 (TM score over 0.5 always indicates the correct model) and C-score of − 1.43 (the highest C-score among the 5 models).

The fifth step of this study was to modify the 3D model by GalaxyWEB server. And after refining the 3D structure, the model was validated by ProSA-web, Molprobity, and ERRAT servers. From the ProSA-web server, the *z*-score shows the overall quality of the model. Its value is displayed in the graph (*z*-score − 3.47), which includes the *z*-scores of all experimental protein chains determined in the recombinant protein structure. The Ramachandran plot showed 79.3% residues in most favored regions, 17.0% residues in additional allowed regions, 1.3% residues in generously allowed regions, and 2.4% residues in disallowed regions. The results of the Ramachandran plot show that the quality of the 3D structure is acceptable. Finally, the ERRAT tool has been used for non-bonded interactions between different types of atoms. The higher the scores in ERRAT, the higher the quality of the 3D structure. The accepted range for the model is higher than 50. The quality score of recombinant protein is 61.842%.

In the final steps of this study, we optimized our recombinant nucleotide sequence to increase the expression of multi-epitope vaccine in *E. coli*. The codon compatibility index (CAI) of the sequence changed from 0.5 to 1.0 and the GC content of the sequence changed from 65.21 to 51.29%.

The results of immunoinformatics analysis showed that the designed multi-epitope construct was a suitable antigen and has the ability to be tested as a vaccine candidate.

## Conclusion

In the present study, using in silico tools, a multi-epitope recombinant vaccine against *B. melitensis* was designed. The purpose of using this method is to save time and money as much as possible. Based on the results of immunoinformatics, the designed construction has virtuous structural, suitable physiochemical characteristics, high antigenicity, good stability, and appropriate solubility, as well as non-allergenic. Therefore, this recombinant vaccine can be used as a suitable candidate to fight against brucellosis infection. But in order to confirm the results of the present studies, the analysis of immunogenicity in in vitro and in vivo tests is very necessary.

## Data Availability

The data that support the findings of this study are available on request from the corresponding author.
